# Sleep quality and the need for recovery among nurses working irregular shifts: A cross-sectional study

**DOI:** 10.3233/WOR-230500

**Published:** 2024-11-08

**Authors:** Uthman Albakri, Nick Smeets, Elizabeth Drotos, IJmert Kant, Andrea Gabrio, Ree Meertens

**Affiliations:** a Department of Health Promotion, NUTRIM School of Nutrition and Translational Research in Metabolism, and Care and Public Health Research Institute (CAPHRI), Maastricht University, Maastricht, The Netherlands; b Department of Public Health, Faculty of Applied Medical Sciences, Albaha University, Albaha, Saudi Arabia; c Department Human Resources. Maastricht University Medical Center (MUMC+), Maastricht, The Netherlands; d Department of Epidemiology, Care and Public Health Research Institute (CAPHRI), Maastricht University, Maastricht, The Netherlands; e Methodology and Statistics, School for Public Health and Primary Care, Faculty of Health, Medicine and Life Sciences, Maastricht University, Maastricht, The Netherlands

**Keywords:** Nurses, hospitals, shift work schedule, circadian rhythm, sleep, fatigue, burnout, need for recovery.

## Abstract

**BACKGROUND:**

Shift work affects the mental and physical health of nurses, yet the effect of working irregular shifts on sleep and its association with the need for recovery is under-explored.

**OBJECTIVE:**

The purpose of this study was to investigate the sleep quality of nurses working irregular shifts, including night shifts, and to determine whether sleep quality is associated with the need for recovery.

**METHODS:**

This cross-sectional study included 405 nurses working irregular shifts. Data were collected using an online questionnaire that included sociodemographic characteristics, the Sleep-Wake Experience List, sleep problems, sleep duration, and the Need for Recovery scale. Data analyses included descriptive statistics, chi-square tests, *t*-tests, logistic and multiple linear regressions.

**RESULTS:**

Nurses who worked irregular shifts had poor sleep quality. Those who also worked night shifts, had significantly poorer sleep quality and experienced more difficulties in daily functioning than those who did not work night shifts. Sleep quality was significantly associated with the need for recovery and this remained so after controlling for confounding variables (*β*= .554, *p* = .001).

**CONCLUSION:**

The findings indicate that in nurses who work irregular shifts, the sleep quality is low. In this group, the sleep quality in nurses who work night shifts is lower than in nurses who do not work night shifts. Furthermore, better sleep quality was associated with lower need for recovery. These findings suggest that improving sleep quality in nurses working irregular shifts may lower their need for recovery, which may improve health, and reduce burnout and sickness absence.

## 1 Introduction

Nurses involved in shift work are required to provide round-the-clock healthcare for their patients. These nurses, especially those who work night shifts, may experience significant negative impacts on their physical and psychological well-being [1]. They also experience a negative impact on their social and family life, and a negative cultural perception of night shifts [2]. Night shift work contributes to sleep deprivation and circadian disruption [3, 4]. Shift work, especially when night shifts are involved, disrupts the normal sleep– wake cycle (i.e., the circadian rhythm), changing hormone levels and leading to less sleep and excessive fatigue, which may contribute to various health problems [5– 8]. Furthermore, some studies suggest that nurses working night shifts have an increased risk for cancer mortality, cardiovascular disease [9], and diabetes [10]. Shift work has been associated with low sleep quality among nurses [11, 12], and different types of shifts have been investigated in relation to sleep quality. For instance, both regular and rotating shifts that include night shifts were found to be associated with low sleep quality [13, 14]. However, the sleep quality of nurses who work irregular shifts, defined as those having irregular shifts that lack a consistent or predictable pattern in terms of both the days and the number of days scheduled within a certain month (hereafter “nurses working irregular shifts”), has not been investigated. According to this definition, rotating shifts are also considered regular shifts, as their patterns are predictable. It is important to note that nurses in the Netherlands frequently work night shifts on an irregular basis, (i.e., not following a predictable pattern), as in, for example, a forward rotating schedule. This is due to most hospital work being scheduled during the day, leading to a lower demand for staff in the night, and making rotating shift schemes for all nurses impossible. This basic irregularity is further aggravated by the labor shortage in the health care sector, the high percentage of nurses on sick leave, and the increase of self-employed nurses. This makes scheduling rosters difficult and rosters are often subject to change. Consequently, night shifts for all nurses are quite irregular. This is particularly relevant, as practitioners and researchers might assume that ‘a few night shifts a month will not hurt.’

Sleep is vital in the body’s recovery process, as it facilitates many restorative functions [15, 16]. Indeed, Kunert found low sleep quality and fatigue to be related [17], and according to Giorgi et al, there is a direct relationship between poor sleep quality and burnout [18]. Surprisingly, sleep quality has received little attention in research as a possible determinant or associated variable to the need for recovery, which is the need to recuperate from work in particular.

### 1.1 Background

Typically, shift work refers to work arrangements outside traditional daytime working hours, including fixed early morning, evening, and night shifts, roster work, and rotating three-shift work [19]. Specifically, night shifts are defined as taking place from 11 p.m. to 6 a.m. [20], although adopted definitions may differ slightly (e.g., 8 p.m. to 7 a.m.) [21].

Knutsson [22] identified different ways in which shift work may impact health, through (1) a mismatching of circadian rhythm; (2) behavioral changes; and (3) disturbed socio-temporal patterns, which could in turn cause gastrointestinal disease, cardiovascular disease, cancer, pregnancy complications, depression, hypertension, and injuries [22, 23]. First, shift work, including night shift work, disrupts individuals’ circadian rhythms [24, 25], which is related to such different health problems as sleep disorders [26], cardiovascular disease, and psychiatric disorders [27]. Second, night shift work may lead to such behavioral changes as dietary and physical habits, like unhealthy snacking and less physical activity during a period of night shifts, which may lead to disease in the long term [24, 28]. Third, night shift work has also been associated with social and family conflicts and satisfaction, as shift work interferes with workers’ personal life [29, 30]. These conflicts may lead to reduced social support and stress [22, 31], sleep deprivation [32], excessive sleepiness [24], fatigue [33, 34], and depression [35].

Overall, sleep quality is determined by a person’s subjective satisfaction with sleep initiation, maintenance, quantity, and sense of refreshment upon waking [36]. Regular shift workers’ sleep quality has been investigated thoroughly, with numerous studies showing that poor sleep quality is prevalent among this population [12, 37, 38]. The sleep quality of nurses who work rotating shifts has also been investigated intensively; a systematic review and meta-analysis concluded that nurses working rotating shifts have poorer sleep quality than those consistently working night shifts [13]. Further, working in irregular three-shift systems has been investigated in the transport and other sectors, but not in the health sector; it was found that working in this system is associated with sleep loss and sleepiness [39]. However, the sleep quality of nurses working irregular shifts has not been investigated thoroughly. Only two studies have specifically examined sleep problems among nurses working these shifts, showing that nurses reported dissatisfaction with their sleep quality [40] and suffered from considerable sleep disturbances [41]. Few studies have examined the association between sleep quality and nurses working different shifts, including night shifts, compared to nurses working day shifts, and the studies in this group did not find a consistent relationship [35, 42–44]. Both Dai et al. [35] and Feng et al. [43] investigated the relationship between different shifts including night shifts and sleep quality among Chinese nurses, and demonstrated that nurses who work night shifts have poorer sleep quality than those who work day shifts. Beebe et al. [42] and Huth et al. [44] investigated the association between nurses’ sleep quality who work different shifts, including night shifts, compared to nurses working day shifts only; neither found a statistically significant difference between the two groups. However, none of these studies clearly state whether the included nurses worked regular or irregular night shifts. Overall, the association between sleep quality and working irregular shifts, including night shifts, among nurses has not been investigated rigorously, and due to the inconsistency in results and paucity of research on this relationship, further investigation is warranted.

The need for recovery is defined as “the need to recuperate from work-induced fatigue, primarily experienced after a day of work” [45] and is similar to concepts like “post-work irritability” [45] and work-related fatigue [46]. The concept of the need for recovery is based on the effort-recovery model [47, 48], which states that the workday generates effort costs that, if sustained over time, result in a variety of emotional, cognitive, and behavioral symptoms. Such symptoms are reversed when effort expenditure is stopped. In the effort-recovery model, physical and psychological job demands may lead to load effects, including a high need for recovery [46, 49]. Studies show that overload occurs when inadequate opportunities for recovery from work are experienced routinely [50], which may result in the development of severe long-term, fatigue-related disorders such as burnout [51], subjective health problems [46], sickness absence [52], and cardiovascular diseases[53].

Sleep is critical for recovery, resource regeneration, and mental and physical wellness [54] and can be viewed as a “moderator” in the effort-recovery model; for example, given that sleep has an effect on work capacity — including work productivity— and work capacity affects the need for recovery [55], sleep may moderate the effect of work capacity on the need for recovery. However, in studies on the need for recovery, sleep has largely been neglected as a possible determinant. In the literature, few studies have investigated the relation between sleep quality/sleep complaints and the need for recovery among the working population [56–58]. These studies found strong positive associations between sleep quality/sleep complaints and need for recovery, as it was found that low sleep quality is related to a high need for recovery; but none of these studies focused on nurses who work *irregular* shifts. To the best of our knowledge, no studies have investigated the relationships between the need for recovery of nurses working irregular shifts and their sleep quality.

To summarize, the adverse effects of night shift work on sleep, health, and well-being have been well-documented in shift workers, including regular shift nurses [59, 60]. Nevertheless, there is still insufficient data on the sleep quality of nurses working irregular shifts, particularly nurses working irregular night shifts. Although sleep quality may play an important role in the process of recovery after work, the association between sleep quality and the need for recovery among nurses working irregular shifts has not yet been investigated. This study aims to fill this critical gap in the literature. By examining the relationship between sleep quality and the need for recovery, this study not only addresses this significant gap but also contributes new insights regarding the impact of irregular shift work on nurses’ recovery processes. The aim of the present study was to investigate (1) the sleep quality of nurses working irregular shifts, (2) whether the sleep quality is worse in nurses working irregular shifts including night shifts than in nurses working irregular shifts but no night shifts, and (3) whether sleep quality is associated with the need for recovery.

## 2 Methods

### 2.1 Design

The present study followed a descriptive cross-sectional design and included nurses who work irregular shifts, including night shifts, as well as those who do not work night shifts. The Guidelines of Strengthening the Reporting of Observational Studies in Epidemiology (STROBE) were used [61].

### 2.2 Setting and participants

Eight hundred and fifty-four nurses registered with an employment contract at Maastricht University Medical Center (MUMC+) in the southern part of the Netherlands were invited to complete an online questionnaire. All nurses were invited to participate in the study. Only interns were excluded, and all other nurses, regardless of their specific roles or departments, were eligible to contribute to our research. A typical day shift is from 7 am to 3 pm, a typical evening shift from 3 pm to 11 pm, while a typical night shift spans from 11 pm to 7 am.

### 2.3 Data collection

The study was carried out between September 2019 and January 2020. A comprehensive online questionnaire was administered that consisted of sociodemographic characteristics, health indicators, and a range of sleep and recovery-related questions. The questionnaire was sent to all nurses via email. Such strategies as having a contact person in each department, polite reminders via email to complete the survey, and presentations in departments were used to encourage completion of the survey.

### 2.4 Ethical considerations

Participation in this study was voluntary, and the research purpose was explained to all participants upon delivery of the questionnaire. All nurses provided written informed consent.

The ethical committee for the Faculty of Health,Medicine, and Life Sciences of Maastricht Univer-sity reviewed and approved the study (FHML/WHC/2019/47).

### 2.5 Measures

Data were collected on sociodemographic characteristics, health, lifestyle behaviors, sleep (Sleep-Wake Experience List (SWEL), sleep problems, sleep duration), and the Need for Recovery Scale (NfR).

#### 2.5.1 Sociodemographic, health, and lifestyle behaviors questionnaire

The following sociodemographic data were collected: age, gender, education level, living situation, years of work experience, average number of working hours per week, whether worked night shifts during the last six months or not (y/n), and average number of night shifts worked per month in the past six months. Items that measured health pertained to perceived general health, treatment for sleep problems, and body mass index (BMI). Lifestyle behaviors were measured using a self-developed questionnaire with questions derived from earlier studies [62, 63]. Five questions solicited information on daily smoking (y/n), alcohol intake (number of glasses per week), engaging in moderate to vigorous physical activity for 150 minutes per week (y/n), frequency of exercise on average per week (1 time, 2 times, 3 times, or more than 3 times), and use of computers or mobile screens within 60 minutes before bedtime (always, occasionally, never).

#### 2.5.2 Sleep outcomes

Sleep quality was assessed using a Dutch version of the SWEL [64]. The 15 questions covered six types of complaints in a 24-hour sleep-wake cycle over the past three months: initiating sleep, maintaining sleep, early awakening, difficulties waking up, waking up tired, and sleepiness during the day. Both the severity and frequency of the complaints were measured and scored on a five-point Likert scale (1 to 5) in reference to the past three months. The complaints were identified as “sleep problems” if described as “rather severe” to “very severe” and as “frequently present” or “always present”. A continuous score was determined by combining the points of the frequency and severity questions for each sleep complaint and weighting them for the total number of questions used to measure each sleep complaint. The results of each complaint were summed, then scaled by 10. The results of all sleep complaints were then summed together and divided by six to get the average continuous score for sleep quality, resulting in a scale ranging from 0 to 11 points, in which a higher score indicated a lower sleep quality.

Two additional survey items concerning sleep problems and daytime functioning were based on the Insomnia Severity Index (ISI) [65]: 1) “To what extent have you experienced problems sleeping in the past two weeks? (Think of difficulty falling asleep, difficulty sleeping, or waking up too early),” and 2) “To what extent have your sleep problems hindered your daily functioning? (Consider daytime fatigue, functioning at work, performing daily tasks, concentration, memory, and mood).” The questions were answered on a 5-point Likert scale: “not at all,” “a little,” “quite,” “much,” and “very much.” Response options “quite,” “much,” and “very much” were coded as “yes,” indicating poor sleep quality while “not at all” and “a little” were coded as “no.” indicating good sleep quality.

Sleep duration was measured by asking the average number of hours participants slept each day. Cut-off points were used to categorize average sleep duration into three groups based on the recommendations of the American Academy of Sleep Medicine and Sleep Research Society, which recommends 7– 9 hours of sleep [66] per night. Sleep duration was categorized as <7 hours, 7– 9 hours, and >9 hours.

#### 2.5.3 Need for recovery

The Need for Recovery scale (NfR) was derived from the Dutch questionnaire Experience and Assessment of Work (VBBA) [67], a scale widely used in the Netherlands [46, 52, 68, 69]. The scale contains 11 dichotomous items (yes/no), concerning the recuperation period after one day of work. Examples of items include, “I find it hard to relax at the end of a working day,” and, “My job causes me to feel exhausted at the end of a working day” [45, 68]. The responses were summed, resulting in a total NfR score ranging from 0 to 11 points. An NfR score (≥6) was defined as a high NfR, while an NfR score (<6) was defined as a low NfR [70].

### 2.6 Sample size calculation

The sample size calculation was performed in accordance with the main research question of the study, which aimed to assess the correlation between sleep quality and the need for recovery. An exact statistical two-tailed test was used to determine the expected number of nurses needed to identify a Pearson’s correlation coefficient of 0.3 under the alternative hypothesis, assuming a type I error rate of 0.05 and a power of 0.9. According to these calculations, done using G*Power software, version 3.1.9.7, the minimum expected number of nurses required is 112 nurses. However, given the exploratory nature of the study and its focus on a relatively under-investigated area, no stopping rule was applied at the time of recruitment and, in the end, 405 nurses were included in the study analysis.

### 2.7 Data analysis

Frequencies and percentages were calculated for categorical variables, and means and standard deviations were computed for continuous variables. Pearson’s *r* correlation coefficients were used to determine the minimum sample size and to assess the presence of a linear association between sleep quality measures and the NfR. Assumptions for analysis of (co)variance (AN(C)OVA), multiple linear regression, and logistic regression were tested. Logistic regression, ANOVA, independent-sample *t*-test, and chi-square analyses were used to compare sociodemographic, health, lifestyle behaviors, sleep duration, sleep quality measures, and the NfR between nurses working night shifts and nurses working no night shifts. For the independent-sample *t*-test, when the p-value for Levene’s test was smaller than the cut-off of.05, we reported the t-value in the equal variance not assumed line. ANCOVAs were used to compare continuous data between groups while controlling for age, gender, and the average number of working hours per.

Further, we performed multiple linear regressions to quantify the associations between the NfR and sleep quality while controlling for multiple confounding variables. In the unadjusted model for the NfR (Model 1), sleep quality, sleep duration, and whether nurses worked night shifts or not in the last six months were entered as predictor variables. In the adjusted model for the NfR (Model 2), age, gender, living situation, general health, being treated for sleep problems, daily smoking, frequency of exercise per week, and average number of working hours per week were entered in a first step in a hierarchical regression as confounding variables. Using the same approach, the sleep quality measurement by the SWEL was replaced by sleep quality as measured by the ISI-based questions. All the statistical analyses were performed using two-tailed tests, and a significance level of 0.05. SPSS v.25 was used to analyze the data.

### 2.8 Validity and reliability

The SWEL and the NfR are validated scales [64, 69], and the scales demonstrated good internal consistency in this study (Cronbach’s *α*= 0.86, *α*= 0.85), respectively.

## 3 Results

### 3.1 General characteristics of the study population

**Fig. 1:**
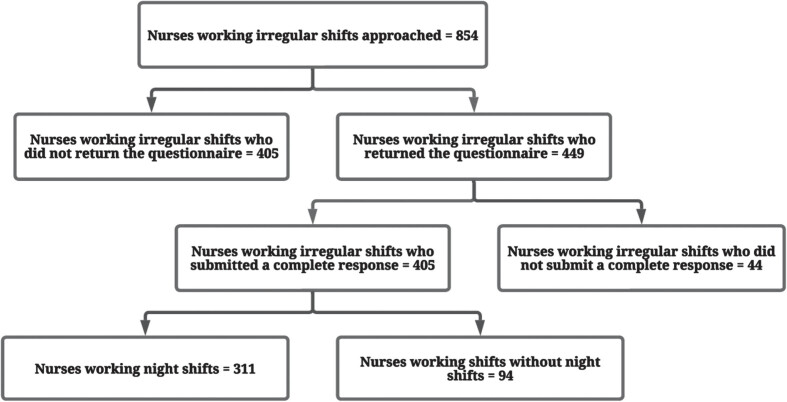
Flow chart showing the participant selection process.

Figure 1 shows the number of participants approached and recruited for the study, those who returned the questionnaire and those who did not, and the number of nurses working irregular shifts including night shifts, as well as those who did not. Of the 854 nurses who received the invitation to participate, 449 nurses returned the questionnaire, with 405 submitting completed response sets retained for the analysis (47%). Table 1 presents the general characteristics of the study population, the majority (69.3%) of whom worked night shifts on an average of 5 nights per month in the past 6 months. The minimum number of night shifts, each lasting 8 hours, was 1 night per month in the last 6 months, while the maximum was 14 nights per month in the last 6 months. The average age of the participants was 40.1 years, ranging from 20 to 66 years old, and the average work experience was 16.9 years. Participants worked an average of 32.2 hours per week, and the majority were female (78.5%). Regarding their health, most nurses had not been treated for sleep problems (95.3%). Furthermore, the nurses’ mean NfR score of 4.7 was considered moderate [70].

**Table 1:** Characteristics of irregular shift nurses stratified by shift types

Variables	Total = 405	Nurses without night shifts (*n* = 94)	Nurses with night shifts (*n* = 311)	*p*-value
**Sociodemographic**				
Age (years) (mean; SD)	40.1; 12.2	44.0; 13.1	39.2; 11.7	.002
Gender (*N*, %)				.919
Male	87 (21.5)	20 (4.9)	67 (16.5)	
Female	319 (78.5)	74 (18.3)	244 (60.2)	
Education level (*N*, %)				.650
Secondary vocational education	132 (30.4)	27 (6.7)	96 (23.7)	
Higher professional education	282 (69.6)	67 (16.5)	215 (53.1)	
Living situation (*N*, %)				.161
Single	56 (13.8)	7 (1.7)	49 (12.1)	
Living with a partner	137 (33.8)	38 (9.4)	99 (24.4)	
Living with partner and child(ren)	184 (45.4)	43 (10.6)	141 (34.8)	
Other living situations	28 (6.9)	6 (1.5)	22 (5.4)	
Work experience (years) (mean; SD)	16.9; 12.5	21.1; 13.5	15.9; 12.0	.001
Average number of working hours per week (mean; SD)	32.3; 5.7	30.9; 6.4	32.7; 5.4	.010
Average number of night shifts worked per month in the past 6 months (mean; SD)	–	0	5.2; 2.3	–
**Health/lifestyle behaviors**				
General health (*N*, %)				.223
Excellent	19 (4.7)	3 (0.7)	16 (4.0)	
Very good	105 (25.9)	18 (4.4)	87 (21.5)	
Good	237 (58.5)	61 (15.1)	176 (43.5)	
Moderate	44 (10.9)	12 (3.0)	32 (7.9)	
Poor	0 (0.0)	0 (0.0)	0 (0.0)	
Being treated for sleeping problems (*N*, %)				.266
Yes	19 (4.7)	2 (0.5)	17 (4.2)	
No	386 (95.3)	92 (22.7)	294 (72.6)	
Body mass index (*N*, %)	.757
Underweight	2 (0.4)	0 (0.0)	2 (0.5)	
Normal weight	203 (51.3)	44 (11.1)	159 (40.2)	
Overweight	140 (35.4)	34 (8.6)	106 (26.8)	
Obese	51 (12.9)	14 (3.5)	37 (9.3)	
Daily smoking (*N*, %)				.686
Yes	46 (10.6)	9 (2.2)	34 (8.4)	
No	362 (89.4)	85 (21.0)	277 (68.4)	
Glasses of alcohol per week (mean; SD)	2.4; 3.2	2.5; 3.3	2.4; 3.3	.703
Moderate to vigorous physical activity for 150 minutes per week (*N*, %)				.165
Yes	303 (74.8)	65 (16.0)	238 (58.8)	
No	102 (25.2)	29 (7.2)	73 (18.0)	
Time of exercise on average per week (for at least 1 hour) (*N*, %)				.004
0 times	150 (37.0)	44 (10.9)	106 (26.2)	
1– 2 times	116 (28.6)	31 (7.7)	85 (21.0)	
3 times or more	139 (34.3)	19 (4.7)	120 (29.6)	
Active use of screens 60 minutes before sleep (*N*, %)				.544
Always	182 (44.9)	43 (10.6)	139 (34.3)	
Occasionally	178 (44.0)	38 (9.4)	140 (34.6)	
Never	45 (11.1)	13 (3.2)	32 (7.9)	
**The Need for Recovery scale**				
Need for Recovery (mean; SD)	4.7; 3.3	4.9; 3.4	4.6; 3.2	.577

As shown in Table 1, nurses working night shifts were significantly younger (*t* (141) = 3.19, *p* = .002) than nurses working shifts, but no night shifts. Nurses working night shifts had a relatively lower mean number of years of work experience (*t* (143) = 3.38, *p* = .001) compared with nurses working shifts, but no night shifts. Nurses working night shifts also worked more hours per week (*t* (130) = –2.60, *p* = .010) compared with the other group. Further, nurses working night shifts exercised significantly more than nurses working shifts, but no night shifts (*X*^2^ (2) = 11.30, *p* = .004). None of the other variables were significantly different across the groups, including the NfR (*t* (389) = 0.55 *p* = .577).

### 3.2 Sleep outcomes

Table 2 shows the mean SWEL score of 3.2 reported by nurses working irregular shifts. The most frequent sleep complaints of the SWEL reported by nurses working irregular shifts were tiredness upon waking up, early morning awakening, and difficulty waking up; problems initiating sleep and maintaining sleep were less common. To get an idea of how the SWEL score of nurses compares to other working populations, we used reference data of the Maastricht Cohort Study, an ongoing cohort study that involves more than 12,000 employees of 45 companies in the Netherlands [71, 72]. The mean SWEL score in the Maastricht Cohort Study for employees who only worked day shifts was 2.2, while employees who worked irregular shifts had an average score 2.6, which shows that sleep complaints were more prevalent among nurses working irregular shifts.

**Table 2:** Sleep outcomes of irregular shift nurses stratified by shift types

Variables	Total	Nurses without night shifts	Nurses with night shifts	*p-*value
**Average total score (0–11) of SWEL (mean; *SD*)**	3.2; 1.4	2.8; 1.3	3.3; 1.4	.007
**SWEL subscale (N** having a sleep problem, %)				
Initiating sleep	28 (7.1)	4 (1.0)	24 (6.1)	.242
Maintaining sleep	33 (8.4)	3 (0.8)	30 (7.6)	.096
Early morning awakening	82 (20.8)	15 (3.8)	67 (17.0)	.330
Difficulty waking up	81 (20.6)	16 (4.1)	65 (16.5)	.398
Tiredness upon waking up	95 (24.1)	20 (5.1)	75 (19.0)	.554
Daytime sleepiness	59 (15.0)	13 (3.3)	46 (11.7)	.804
**ISI-based questions** (*N*, %)				
Sleep problems, including *difficulty falling asleep*, *difficulty sleeping,* and *waking up too early*				.338
Yes	162 (41.1)	33 (8.4)	129 (32.7)	
No	232 (58.9)	58 (14.7)	174 (44.2)	
Hindering of daily functioning				.009
Yes	101 (25.6)	14 (3.6)	87 (22.1)	
No	293 (74.4)	77 (19.5)	216 (54.8)	
**Average number of hours of sleep** (*N*, %)				.629
<7 hours	120 (30.5)	24 (6.1)	96 (24.4)	
7–9 hours	272 (69.2)	66 (16.8)	206 (52.4)	
>9 hours	1 (0.3)	0 (0.0)	1 (0.3)	

The analysis of the ISI-based questions of the current study showed that more than half the participants had sleep problems, including difficulty falling asleep, difficulty sleeping, and waking too early, and the majority of participants had difficulties in their daily functioning. Most participants slept the recommended hours (7–9), while fewer than a third of participants slept less than recommended.

Table 2 also presents the results of sleep outcomes according to shift type. The mean SWEL score of 3.3 for nurses working night shifts is higher than the mean SWEL score of 2.8 nurses working shifts, but no night shifts, indicating that nurses working night shifts had poorer sleep quality (*t* (393) = –2.72, *p* = .007). After adjusting for age, gender, and the average number of working hours per week as covariates, a significant effect of shift type remained (*F* (1, 387) = 3.93, *p* = .048, *η*^2^ = 0.01). However, there were no significant differences between the two groups in the specific components (sleep complaints) of the SWEL.

For the ISI-based measurement of sleep problems, there was no significant difference between the two groups *χ^2^* (1, *N* = 394) = .919, *p* = .338). However, there was a significant difference in the hindrance of daily functioning *χ*^2^ (1, *N* = 394)=6.75, *p* = .009), indicating that nurses working night shifts perceived a greater difficulty in their daily functioning than nurses working shifts, but no night shifts. Logistic regression results indicated that, after controlling for age, gender, and the average number of working hours per week, shift type was not significantly associated with sleep problems (OR = 1.33; 95% CI: 0.81– 2.18, *p* = .259). However, shift type remained significantly associated with the hindrance of daily functioning, where the odds were almost 2.18 times higher (OR = 2.18; 95% CI: 1.15 – 4.11, *p* = .016) for nurses working night shifts compared to nurses working shifts, but no night shifts. There is no significant difference between the two groups in regards to the average number of hours sleeping.

### 3.3 Association between the need for recovery and sleep quality

The correlation analysis between the NfR and sleep quality (as measured by the composite SWEL score) showed that the NfR and sleep quality were correlated positively (*Pearson*’*s r* = .56, *p* = .001). Table 3 presents the results of the multiple linear regression for the association between the NfR and sleep quality after controlling for potential confounding covariates. In the unadjusted model (Model 1) and the adjusted model (Model 2), sleep quality was associated positively and significantly with the NfR, indicating that the lower the sleep quality the higher the NfR (*β*= .595; 95% CI: 1.17–1.56, *p* = .001; *β*= .554; 95% CI: 1.06–1.48, *p* = .001). Furthermore, in Model 1, nurses working night shifts were more likely to have a low NfR (*β*= –.106; 95% CI: –1.51 – –0.18, *p* = .012). However, after controlling for confounding variables (Model 2), the association between working night shifts and NfR was attenuated and no longer significant (*β*= –.076; 95% CI: –1.28–0.08, *p* = .082). Further analysis revealed that changes in the association’s significance were primarily due to the variable of general health. Further, sleep duration was not significantly associated with the NfR in the two models.

**Table 3:** Multiple linear regression results with sleep quality (SWEL), working night shifts (yes/no) and sleep duration as predictor variables and the need for recovery as an outcome variable

Variables	Model 1			Model 2		
	*β*	95% CI	*p-*value	*β*	95% CI	*p-*value
Sleep quality (composite SWEL score)	.595	1.18– 1.56	.001	.554	1.07– 1.48	.001
Working night shifts during last six months (No/Yes)	–.106	–1.51 – –0.19	.012	–.076	–1.28– 0.08	.082
Sleep duration	.081	–0.01–0.50	.055	.041	–0.15–0.40	.364

Model 1: unadjusted model. Model 2: adjusted for age (continuous), gender, living situation, general health, being treated for sleep problems, daily smoking, frequency of exercise per week, and average number of working hours per week (continuous).

The correlation analysis between the NfR and sleep quality (as measured by the ISI based questions) showed that sleeping problems as well as hindrance in daily functioning because of sleep problems were significantly correlated with NfR (*Pearson*’*s r* = .31, *p* = .001, and *r* = .38, *p* = .001, respectively). Table 4 presents the results of the association between sleep quality (as measured by the ISI based questions) and the NfR after controlling for potential confounding covariates. In the unadjusted model (Model 1) and the adjusted model (Model 2), it was found that both sleep problems and hindrance in daily functioning because of sleep problems were associated significantly with the NfR. This finding indicates that nurses working irregular shifts with more sleep problems and hindrances in daily functioning were more likely to have a higher NfR (Model 1: *β*= .163; 95% CI: 0.34–1.85, *p* = .004, and *β*= .319; 95% CI: 1.59–3.26, *p* = .001; Model 2: *β*= .137; 95% CI: 0.16–1.67, *p* = .017, and *β*= .263; 95% CI: 1.16–2.84, *p* = .001, respectively). Further, working night shifts or not and sleep duration were not associated with the NfR in the two models for the ISI-based questions.

**Table 4:** Multiple linear regression results with sleep quality (ISI-based questions), working night shifts (yes/no) and sleep duration as predictor variables and need for recovery as an outcome variable

Variables	Model 1			Model 2		
	*β*	95% CI	*p-*value	*β*	95% CI	*p-*value
Sleep problems including *difficulty falling asleep*, *difficulty sleeping,* and *waking up too early*	.163	0.34–1.85	.004	.137	0.16–1.68	.017
Hindering of daily functioning	.319	1.59–3.26	.001	.263	1.16–2.84	.001
Working night shifts during last six months (No/Yes)	–.072	–1.31–0.16	.126	–.056	– 1.21– 0.30	.241
Sleep duration	.084	–0.03–0.55	.080	.019	–0.25–0.36	.712

Model 1: unadjusted model. Model 2: adjusted for age (continuous), gender, living situation, general health, being treated for sleep problems, daily smoking, frequency of exercise per week, and average number of working hours per week (continuous).

## 4 Discussion

This study explored the sleep quality of nurses who work irregular shifts and investigated the associations between the need for recovery and sleep quality. The consequences of working irregular shifts have hardly been investigated before. The findings of the study revealed that nurses working irregular shifts including night shifts had poorer sleep quality than nurses working irregular shifts, but no night shifts. This difference in sleep quality also remained after controlling for factors like gender, age and perceived overall health. This finding aligns with those of two previous studies, which demonstrate that nurses who work night shifts had poorer sleep quality compared to nurses working day shifts only [35, 43]. However, two other studies did not find any association between working night shifts and sleep quality [42, 44]. Furthermore, it should be noted that all these previous studies did not clearly state whether the included nurses worked irregular or regular night shifts. Further, analysis of the ISI-based questions showed that nurses working night shifts were more prone to having difficulties with daily functioning compared to nurses working shifts, but no night shifts. This finding is consistent with Beebe et al.’s study [42], which found that night shift nurses had more difficulty functioning during the day. Ljevak et al. [73] showed also that, in general, shift work— including night shifts— increased the amount of stress and difficulty in coping with shift work and reduced levels of life enjoyment.

In general, this study found that nurses working irregular shifts had poorer sleep quality than the referenced working populations (daytime workers as well as irregular shift workers included in the Maastricht Cohort Study). Our results suggest that working irregular shifts is associated with lower sleep quality among nurses and that nurses working irregular shifts were particularly prone to tiredness upon waking up, early morning awakening, and difficulty waking up, as demonstrated by the SWEL.

Overall, as mentioned in the introduction, Knutsson [22] suggests that working night shifts may result in circadian rhythm disruption. The study’s findings suggest that nurses perceive challenges related to sleep quality in connection with night shifts. This connection likely stems from the mismatch of circadian rhythm that results from working irregular night shifts. However, we cannot exclude the possibility that the other pathways Knutsson mentions (behavioral changes and disturbed socio-temporal patterns) play a role as well.

Furthermore, we found that sleep quality is associated with the need for recovery, showing that nurses working irregular shifts with lower sleep quality had a higher need for recovery after work. To the best of our knowledge, this relation has not been found before in employees working irregular shifts. The study’s finding of a strong association between sleep quality and the need for recovery complements Knutsson’s theory, emphasizing how the mismatch of circadian rhythm may impact sleep, which, in turn, may affect overall recovery and health. Furthermore, this association suggests that sleep quality can be viewed as a “moderator” in the effort-recovery model, which may moderate the effects of working irregular shifts on the need for recovery after work. This finding is in line with studies that assessed the association between sleep quality or sleep-related problems with the need for recovery among different working populations, demonstrating that low sleep quality or sleep-related problems are associated with high need for recovery among different workers [56–58]. Further, we found that while there is no difference in the need for recovery among nurses working night shifts and nurses working also irregular shifts but no night shifts, there is a difference in the sleep quality between the two groups. This may be explained by the “healthy worker” effect. Nurses who work night shifts may suffer from sleep problems over time. When these problems become chronic and the need for recovery increases, some nurses may quit their jobs or arrange for as few night shifts as possible. This results in that, eventually, only the nurses who can handle working night shifts will still work these shifts. Our finding that nurses who worked irregular night shifts were younger and had less work experience may support this “healthy worker” explanation for the lack of difference in need for recovery between the two groups (a secondary selection effect). Overall, the present study finds that sleep quality is strongly associated with the need for recovery among nurses working irregular shifts, also after controlling for the confounding variables.

### 4.1 Strengths and limitations

To the best of our knowledge, our study is among the few that assess the sleep quality of nurses working irregular shifts, comparing those who work night shifts with those who do not, and it is the first to assess the association between sleep quality and the need for recovery in this population. This study has several strengths. First, a relatively large sample of nurses who work irregular shifts was included. Second, we used validated questionnaires for our main measures of sleep quality and the need for recovery.

Still, this study does have several limitations worthy of mention. First, causal inferences cannot be made due to the cross-sectional and exploratory nature of the design. Second, there are currently no established cut-off points to categorize cases with markedly poor sleep quality in the sleep quality questionnaire used. Third, although the sample size of this study is relatively large, it is limited to the nurse population of a specific Dutch hospital, which lessens the ability to generalize the findings. Lastly, we cannot rule out the possibility that there are differences in the need for recovery in relation to other confounders, such as work-related factors, working conditions (e.g., workload), and job demands (e.g., work pressure), which have been found to be associated with the need for recovery in general [74]. However, we questioned the hospital regarding the differences between the two groups in regard to work characteristics, including work content, job title, and job demand, and the response was that there are no differences between the two groups regarding work content and work characteristics and that they do not expect any differences regarding job demands between the two groups.

### 4.2 Implications for practice and research

This study shows that nurses who work irregular shifts suffer from low sleep quality; their sleep quality seems even worse than that of other groups of irregular workers (reference group Maastricht Cohort Study). This suggests that, perhaps contrary to what one would expect, even a night shift now and then may have detrimental effects on sleep quality. Our study also shows that nurses who work irregular shifts including night shifts have poorer sleep quality than those who do not work any night shifts. The study also found a positive relationship between sleep quality and the need for recovery, indicating that lower sleep quality was associated with a higher need for recovery in nurses working irregular shifts. A high need for recovery has been shown to be a strong predictor of impaired well-being [68], subjective health complaints [44], prolonged fatigue [75], burnout [51], and sickness absence [52]. Therefore, an important practical implication of this finding is that a high need for recovery among nurses may be prevented partly by interventions that improve their sleep quality. There are no specific evidence-based coping strategies for nurses who work irregular shifts to improve their overall sleep quality. However, strategies have been suggested for those who work shifts in general, and these strategies may work for nurses working irregular (night) shifts as well. Such strategies include getting plenty of sleep prior to a shift, and resetting after a set of night shifts, e.g., taking a 90-minute or 180-minute nap (one or two full sleep cycles) and then re-aligning with daily sleep cues by going outside into bright light, socializing, and going to bed as near to the usual time as possible [76–78]. Regarding future studies, research should investigate the coping mechanisms that nurses use to manage the challenges of irregular shift work and explore the development of personalized sleep hygiene programs. Such studies could provide invaluable insights into effective strategies to support the well-being of nurses working irregular shifts.

## 5 Conclusion

The present study is one of the few studies that shed light on possible consequences of irregular shift work, a topic that has largely been neglected in the literature. Our findings reveal several key insights. First, our findings show that nurses who work irregular shifts suffer from low sleep quality. Second, our results show that nurses working night shifts had poorer sleep quality than those who work shifts, but no night shifts. Third, this study is the first to find evidence that sleep quality is significantly associated with the need for recovery of nurses working irregular shifts. It is suggested that low sleep quality for shift nurses may lead to high need for recovery, which may affect the future health status. Therefore, it is important to assess in future research whether sleep quality in nurses working irregular shifts can be improved by either optimizing irregular shifts or by using certain sleep hygiene strategies.
